# Quantitative trait loci for variation in immune response to a Foot-and-Mouth Disease virus peptide

**DOI:** 10.1186/1471-2156-11-107

**Published:** 2010-12-07

**Authors:** Richard J Leach, Susan C Craigmile, Sara A Knott, John L Williams, Elizabeth J Glass

**Affiliations:** 1 The Roslin Institute and Royal (Dick) School of Veterinary Studies, University of Edinburgh, Roslin Biocentre, Roslin, Midlothian, EH25 9PS, UK; 2 Institute of Evolutionary Biology, University of Edinburgh, Ashworth Laboratories, King's Buildings, West Mains Road, Edinburgh, EH9 3JT, UK; 3 Parco Tecnologico Padano, via Einstein, Lodi, 26900 Italy

## Abstract

**Background:**

Infectious disease of livestock continues to be a cause of substantial economic loss and has adverse welfare consequences in both the developing and developed world. New solutions to control disease are needed and research focused on the genetic loci determining variation in immune-related traits has the potential to deliver solutions. However, identifying selectable markers and the causal genes involved in disease resistance and vaccine response is not straightforward. The aims of this study were to locate regions of the bovine genome that control the immune response post immunisation. 195 F2 and backcross Holstein Charolais cattle were immunised with a 40-mer peptide derived from foot-and-mouth disease virus (FMDV). T cell and antibody (IgG1 and IgG2) responses were measured at several time points post immunisation. All experimental animals (F0, F1 and F2, n = 982) were genotyped with 165 microsatellite markers for the genome scan.

**Results:**

Considerable variability in the immune responses across time was observed and sire, dam and age had significant effects on responses at specific time points. There were significant correlations within traits across time, and between IgG1 and IgG2 traits, also some weak correlations were detected between T cell and IgG2 responses. The whole genome scan detected 77 quantitative trait loci (QTL), on 22 chromosomes, including clusters of QTL on BTA 4, 5, 6, 20, 23 and 25. Two QTL reached 5% genome wide significance (on BTA 6 and 24) and one on BTA 20 reached 1% genome wide significance.

**Conclusions:**

A proportion of the variance in the T cell and antibody response post immunisation with an FDMV peptide has a genetic component. Even though the antigen was relatively simple, the humoral and cell mediated responses were clearly under complex genetic control, with the majority of QTL located outside the MHC locus. The results suggest that there may be specific genes or loci that impact on variation in both the primary and secondary immune responses, whereas other loci may be specifically important for early or later phases of the immune response. Future fine mapping of the QTL clusters identified has the potential to reveal the causal variations underlying the variation in immune response observed.

## Background

Infectious disease of livestock continues to be a cause of substantial economic loss and has adverse welfare consequences, even in well managed agricultural systems [1]. In addition, even with stringent bio-security, there are incursions of "exotic" diseases (e.g. the recent Foot-and-Mouth Disease (FMD) outbreaks within the E.U. [2]). Current interventions against infectious disease include anthelminthics, antibiotics and other chemicals as well as vaccination, although for many endemic and exotic diseases there are limited appropriate and effective controls. Thus alternative solutions for disease control are needed. Breeding for disease resistance together with more effective vaccines have the potential to deliver solutions.

There is considerable variation among individuals in the response to infectious disease and vaccination, a significant proportion of which can be shown to be genetic [1]. It is clear that the wide range in immune responsiveness and disease resistance found within livestock populations is controlled by many genes. Many candidates genes have been identified that may influence the immune response, including the Major Histocompatibility Complex (MHC), however, the relative contribution of the MHC and non-MHC loci to the wide variation in immune-related traits is only beginning to be explored. Identifying and understanding the role of different polymorphic loci in immune-related traits may lead to the identification of selectable markers for disease resistance, and may also suggest new host targets to improve vaccine efficacy.

Identifying the causal genes involved in disease resistance and vaccine response is not straightforward. Phenotypes for these traits often require complex measurements and are expensive to collect. Often field data has been used which has inherent limitations and is subject to variation caused by considerable environmental influences. The actual correlates of protection are often unknown and likely to be a complex combination of innate and acquired immunity. Many diseases, such as mastitis can be caused by distinct pathogens which result in different responses that are likely to be under different genetic controls [3]. The causal pathogen is often not identified in field studies. In addition, data collected may consist of single time points and thus cannot account for variation in the kinetics of immune responses. In order to address some of these issues and to explore the contribution of the MHC and other loci to variation in immune responses, a cross-bred cattle population was immunised with a relatively simple 40-mer peptide derived from the FMD virus (FMDV). The peptide was used primarily as a model for eliciting an immune response, which allowed a whole genome scan to be conducted using microsatellite markers distributed across the bovine genome.

The FMDV peptide (FMDV15) used in this study consists of two sections of the VP1 protein located on the FMDV capsid, together encompassing the major neutralising antibody sites. VP1 is one of four FMDV structural proteins (VP1-4) and contains a loop structure that is present on the surface of the virus, which is particularly immunogenic [4]. Protection against FMD is generally believed to relate to the levels of neutralising antibody and has been correlated with IgG1 and IgG2 levels [5,6]. In addition it has been shown that cell mediated responses also play a role in protection against FMDV [7,8]. We have previously shown that there is considerable variation in the immune response to this and related peptides as well as variation in the protection against FMDV challenge [9,10]. Although our previous studies revealed that polymorphisms in loci within the bovine MHC (BoLA), particularly the class II *DRB3 *gene, accounted for some of the variation in immune response to FMDV [9-11], it seems likely that other genetic factors are also important. Considerable animal to animal variation has been reported in the context of FMDV challenge and immunisation with a variety of vaccine constructs including peptides. However the role of host genetics in variation of the response to immunisation has not been generally considered: there has only been one study which suggested that host genetic factors might play a role in response to vaccination with inactivated FMDV vaccines [12].

The work reported in this paper analysed the variability in the kinetics of FMDV15 specific IgG1, IgG2 and T cell response during a primary and secondary response following peptide immunisation of 195 second generation Charolais Holstein backcross heifers. The variations in responses to the peptide were correlated with 165 microsatellite markers distributed across the bovine genome to identify QTL controlling variation in the immune response. These animals were part of a larger experimental population, the Roslin Bovine Genome (RoBoGen) herd, and our earlier research on immune traits in the RoBoGen herd had demonstrated that there was a genetic component for the IgG response to a Bovine Respiratory Syncytial Virus (BRSV) vaccine, with a peak heritability of 0.36 [13]. Further, the T cell proliferative response to a mastitis causing pathogen showed significant sire effects [14]. In addition, meat and milk quality QTL [15] as well as coat colour QTL [16] have been detected in this population. In this first study to map QTL for variation in the immune response following a standardised controlled immunisation, regions of the bovine genome were identified that control a proportion of variance of the immune response to the FMDV15 peptide.

## Results

The 195 female F2, Holstein back-cross and Charolais back-cross animals of the "RoBoGen" population were immunised with the FMDV15 peptide and the resulting antibody and T cell responses were measured across time. The T cell responses to a T cell mitogen, Concanavalin A (ConA) were also measured across time. The whole herd (males and females in the F0 to F2 generation, in total 984 animals) was genotyped with 165 microsatellite markers.

### Kinetics of responses to immunisation

**Figure 1:**
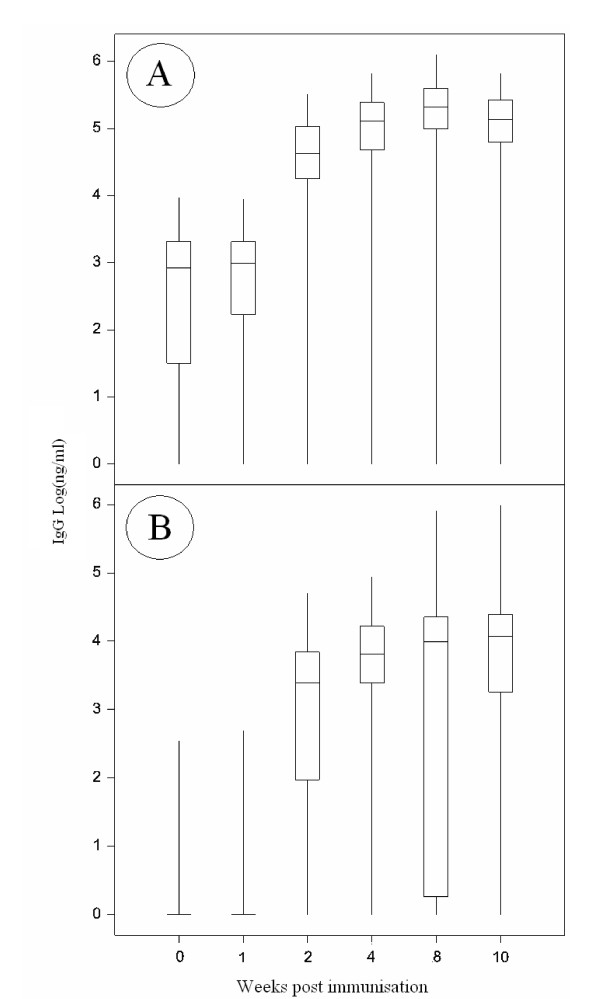
**Variation in IgG isotype responses**. FMDV15 peptide specific IgG1 (A) and IgG2 (B) levels following immunisation at weeks 0 and 6 of 195 female cattle. Median (central horizontal line), quartiles (outer horizontal lines) and range (outer vertical lines) shown. Over 90% of the animals at week 0 and week 1 showed a very low IgG2 response. Thus the median and quartiles tend to zero at these weeks. All data is log_10 _transformed.

**Figure 2:**
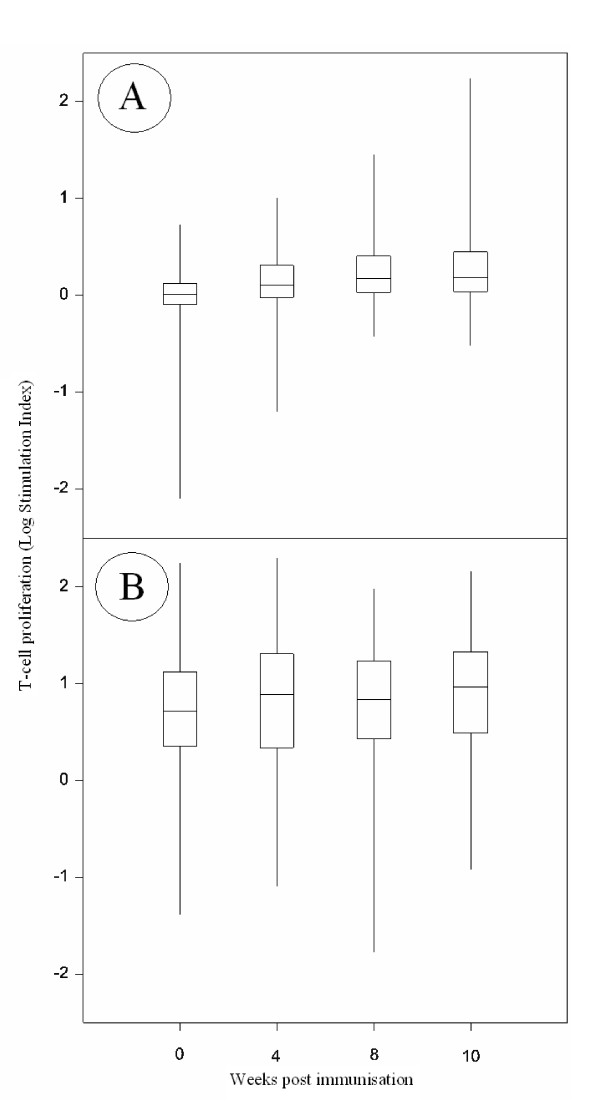
**Variation in T cell proliferation**. Box plots depicting T cell proliferation (stimulation index) across time following immunisation with FMDV peptide. 2 ug/ml FMDV15 peptide (A), and 10 ug/ml Con A (B). Median (central horizontal line), quartiles (outer horizontal lines) and range (outer vertical lines) shown. FMDV peptide administered at week 0 and 6. All data has been log_10 _transformed.

A humoral and cell-mediated response to the FMDV15 peptide was detected in all animals (Figure 1 and 2). From week 2, responses were significantly different from week 0 for anti FMDV15 peptide specific IgG1 and IgG2, and from week 4 for the T cell proliferative response to the FMDV15 peptide. Following a second immunisation, the IgG1 and T cell responses to the FMDV peptide increased significantly (p < 0.01). The mean values for the IgG1 responses (Standard Deviation, SD) for weeks 0, 1, 2, 4, 8, 10 and AUC (area under the curve, to provide a single trait that reflected the overall response, see methods.) were 1.2 (1.4), 1.3 (1.4), 69.7 (66.1), 167 (144.4), 291.7 (269.2), 176.1 (141.6) and 1658.4 (1381.3) μg/ml respectively. The IgG2 responses for weeks 0, 1, 2, 4, 8, 10 and AUC were 0.01 (0.04), 0.01 (0.05), 5.6 (8.8), 13.1 (17.3), 24.3 (64.7), 23.5 (71.7) and 144.4 (302.3) μg/ml respectively. The means for the T cell stimulation index of response to the FMDV peptide at weeks 0, 4, 8, 10 and AUC were 1.131 (0.58), 1.8 (1.5), 2.6 (3.7), 4.6 (14.6), 21.9 (27.3) SI. Correlations between the time points and between traits (Additional File 1) revealed that there was a high correlation between the antibody levels at week 0 and 1 (r^2 ^= 0.88, p < 0.0001 for IgG1; r^2 ^= 0.61, p < 0.0001 for IgG2). Most of the significant correlations were positive. The early IgG1 response (week 2) correlated with later responses at week 4 (r^2 ^= 0.31, p < 0.0001) and 8 (r^2 ^= 0.26, p < 0.0001) and the pre-boost response at week 4 correlated with the post-boost response at week 10 (r^2 ^= 0.69, p < 0.0001). In addition responses at weeks 8 and 10 were also highly correlated (r^2 ^= 0.38, p < 0.0001). The IgG2 response showed similar correlations in so far that earlier responses correlated with later responses, with the strongest correlations (r^2 ^> 0.4, p < 0.0001) seen between weeks 2 and 4, weeks 4 and 8, and weeks 8 and 10. Similarly, the pre-boost T cell response to the FMDV15 peptide at week 4 correlated with the post-boost response at weeks 8 and 10 (r^2 ^= 0.224 and r^2 ^= 0.155; p < 0.0001), with the highest correlation between weeks 8 and 10 (r^2 ^= 0.57, p < 0.0001). The ConA T cell responses correlated moderately across all time points with r^2 ^> 0.2, except between weeks 0 and 4 which had an r^2 ^= 0.12 (all correlations had a significance of at least p < 0.0001).

Furthermore, correlations were found between some of the different trait types (Additional File 1). The IgG1 and IgG2 responses were moderately correlated at the same weeks from 2 to 10 (r^2 ^at least 0.14, all values p < 0.0001). No significant correlations were seen between the anti FMDV15 T cell response and IgG1 responses. However, some weakly significant correlations were seen between the T cell response and the IgG2 response, with one highly significant correlation at week 8 (r^2 ^= 0.14, p < 0.0001). In addition the T cell response to the FMDV peptide correlated with the ConA response, with the strongest correlations seen at the same weeks 4, 8 and 10 (r^2 ^> 0.43, p < 0.0002).

### Analysis of fixed and random effects

As reported above considerable variation was observed between animals for all three immune parameters, IgG1, IgG2 levels and T cell proliferation (Figure 1 and 2A). The greatest variation among animals was seen in the IgG2 response at week 8, which was significantly greater than the variation seen at any other time point throughout the IgG1 or IgG2 response (p < 0.001). In addition, the proliferative response to ConA varied among animals and across time points following immunisation (Figure 2B). The T cell proliferative response to the FMDV15 peptide (Figure 2) showed less variation among animals across the time points than the T cell proliferation to Con A. The skew (change in low to high responses, Figure 2) in the T cell response to the FMDV15 peptide changed over time, from a negative skew to a positive skew as more animals made a response in the later time points.

When week was included as an interaction within the REML model (data not shown), cohort was significant for both the T cell response to the FMDV peptide (p < 0.001) and the T cell response to Con A (p < 0.001). Further, weight at vaccination was found to be significant for both T cell response to the FMDV peptide (p = 0.015) and the T cell response to Con A (p = 0.019), with lighter animals having higher T cell responses. Age at initial vaccination was only significant for the IgG2 responses to the FMDV peptide (p = 0.005), with older animals having higher IgG2 levels.

When the traits were analysed week by week (Additional File 2) cohort was significant throughout for each trait, with the exception of the IgG2 response, for which cohort was only significant twice throughout the time course. The effects of age and weight also differed: age at vaccination was significant from weeks 0 and 1 for IgG1 responses and only at week 1 for IgG2 responses. No significant age effect was found for either of the T cell proliferation responses. Weight was found to be significant for T cell proliferation to the FMDV peptide at week 8 post vaccination (p = 0.049). Line (F2, CB1, and HB1) was not a significant factor for any traits.

Sire and dam effects varied throughout the study (Additional File 2). However, some sires were nested within lines. No significant sire effects were seen for the IgG1 responses. Some dam effects were detected; however most dams only had one calf. The IgG2 response had no significant dam effects whereas sire was significant from weeks 2 to 10 inclusive (all p < 0.05, with weeks 8 and 10 being p < 0.005). The T cell proliferation responses showed no significance association with sire or dam, with the one exception of a dam effect at week 0 for the Con A response (p = 0.005).

### Overall QTL Results

A total of 77 QTL were identified for all 4 of the trait types (IgG1 and IgG2 concentrations; T cell proliferation to Con A and FMDV15 peptide) studied (Table 1). Of these, 11 were above the 1% chromosome significance threshold (but below the genome wide 5% significance threshold), 2 were above the genome wide 5% significance threshold (but below the genome wide 1% significance threshold) and 1 was above the 1% genome wide significance threshold. The initial linkage association analysis revealed 54 QTL, and a further 23 were located by fitting each QTL as a background effect. The average (mean) F-value for QTL above the 1% chromosome threshold was 6.40 (standard deviation (SD) 0.38), the average F-value for QTL above the 5% chromosome threshold was 4.52 (SD 0.38). The majority of the confidence intervals (CI) (Additional File 3) were large with an average of 72.8 cM calculated across all QTL above the 5% chromosome wide significance level. The CI reflected the estimated QTL effect size, the number of animals used in the study and the density of the markers used. The average phenotypic variance (corrected for fixed effects) accounted for in this study across all QTL above the 5% chromosome wide threshold was 5.8%. A total of 46 significant additive effects were found (Table 1). In 18 of these instances the Charolais allele increased the traits value, whereas the remaining 28, the Holstein allele increased the traits value. Dominance effects were significant in 42 QTL, showing if the alleles were dominant or recessive (see methods).

**Table 1:** All QTL located in this study

Chr^1^	Trait^2^	cM^3^	F^4^	a^5^	d^5^	Var%^6^
2	IgG1_week_10	38	5.60	-0.21**	0.26**	5.71
3	IgG2_week_2	58	5.93	0.66**	0.40	6.03
4	IgG1_week_1	31	5.35	-0.63**	0.34	5.46
4	IgG1_week_8	20	5.17	0.12	0.17**	1.18
4	IgG1_week_10	27	4.92	0.23	-0.47*	5.05
4	IgG1_AUC	28	6.37*	0.11	-0.43*	6.64
4	SI_FMDV_Week_0	67	4.79	0.00	-0.14**	4.92
5	SI_CA_Week_0	68	8.10*	-0.13*	0.24**	8.05
5	SI_CA_Week_0	100	5.76	0.22**	-0.14	6.11
5	SI_CA_Week_10	76	5.26	-0.05	0.23**	5.38
5	SI_CA_10 ug_AUC	69	5.89	-0.06	0.17**	6.04
6	SI_CA_Week_0	30	8.69**	0.26***	0.08	8.59
6	SI_FMDV_Week_8	59	6.49	0.14**	-0.08	6.56
6	SI_FMDV_Week_8	133	7.35*	0.04	-0.20***	7.51
6	SI_FMDV_Week_10	3	6.61	0.12**	-0.15*	6.67
6	SI_FMDV_AUC	2	7.91*	0.10***	-0.07	7.96
6	SI_FMDV_AUC	133	6.21	0.03	-0.13**	6.56
7	IgG2_week_8	0	6.24	-0.85***	0.13	6.58
7	SI_CA_Week_10	0	4.89	0.05	0.28**	5.02
7	SI_FMDV_Week_4	54	5.45	-0.11**	-0.09	5.62
9	IgG1_week_0	40	5.29	-0.2948*	0.35	5.41
9	SI_FMDV_Week_10	63	4.92	0.05	-0.23**	5.05
11	IgG1_week_0	0	5.26	0.25	-0.61*	5.38
12	IgG2_week_8	17	5.44	-0.03	1.24***	5.56
13	IgG1_week_1	0	5.08	0.04	-0.54**	5.20
14	IgG2_week_0	2	4.85	0.12	-0.28**	4.98
15	IgG2_week_8	13	5.16	-0.08	1.03**	5.28
15	IgG2_AUC	27	5.39	-0.08	0.67**	5.51
16	IgG2_week_2	76	6.51	-0.71***	-0.16	6.58
16	SI_FMDV_AUC	64	4.42	-0.06*	-0.07*	4.61
18	SI_CA_Week_10	22	7.02*	-0.20***	0.10	7.06
18	IgG2_week_1	57	5.23	-0.10	0.34**	5.40
19	IgG2_week_2	28	4.93	-0.50*	-0.62*	5.06
19	IgG2_week_4	34	5.64	-0.73***	-0.32	5.75
19	IgG2_week_8	50	5.28	-0.81**	-0.25	5.63
19	SI_CA_Week_8	32	8.57*	0.25***	-0.07	8.48
19	SI_CA_Week_10	32	4.54	0.15*	-0.13	4.67
19	SI_CA_10 ug_AUC	28	4.42	0.13**	-0.01	4.61
20	IgG1_week_2	28	4.74	-0.03	0.48**	4.87
20	IgG1_week_4	36	7.86*	0.20**	0.35**	7.83
20	IgG1_week_8	9	6.49*	0.19	0.64**	6.56
20	IgG1_week_10	23	5.37	0.09	0.38**	5.48
20	IgG1_AUC	20	10.07***	0.12	0.39***	9.82
20	IgG2_week_4	32	7.48	0.52**	0.65*	7.48
20	IgG2_week_8	25	8.46	0.76**	0.87*	8.38
20	IgG2_week_10	31	6.23	0.33	0.82**	6.31
20	IgG2_AUC	31	8.5*	0.40**	0.67**	8.41
20	SI_FMDV_Week_0	61	7.95*	0.05	-0.18***	7.92
21	IgG1_week_2	69	4.96	-0.22*	0.38*	5.09
21	SI_CA_10 ug_AUC	84	4.10	-0.14**	-0.01	4.28
23	IgG1_week_0	78	5.11	-0.19	-0.54**	5.24
23	IgG1_week_1	65	5.83	-0.13	-0.87***	5.92
23	IgG1_week_2	39	4.16	0.37	-0.53	4.31
23	IgG1_week_10	18	5.25	0.37**	0.03	5.37
23	IgG1_AUC	18	5.34	0.29**	0.06	5.46
23	IgG2_week_4	0	5.45	0.63**	-0.18	5.57
23	IgG2_week_8	5	5.85	0.88***	-0.18	5.94
23	IgG2_week_10	33	4.08	1.02**	0.77	4.23
23	IgG2_AUC	6	6.61*	0.66***	-0.18	6.67
24	IgG1_week_8	35	7.83**	0.58***	0.32	7.80
24	IgG1_week_10	33	4.18	0.27**	0.06	4.32
24	IgG1_AUC	33	6.23	0.25***	0.12	6.31
24	IgG2_week_10	21	5.09	0.69**	0.61	5.32
24	SI_CA_Week_4	34	5.46	-0.05	0.28**	5.63
25	IgG1_week_0	0	4.48	0.34*	0.28	4.62
25	IgG1_week_1	0	4.88	0.29*	0.35*	5.01
25	IgG1_week_2	33	4.42	0.05	-0.35**	4.66
25	IgG2_week_1	15	4.48	0.18**	-0.11	4.62
25	IgG2_week_2	34	4.65	-0.14	-0.73**	4.78
25	IgG2_week_4	34	4.85	-0.14	-0.70**	4.98
25	IgG2_AUC	34	5.28	-0.04	-0.62**	5.40
25	SI_CA_Week_10	17	5.79	-0.11*	0.19**	5.89
26	IgG1_week_1	21	4.90	-0.30*	0.35	5.25
27	IgG2_week_4	7	4.61	-0.57**	-0.33	4.75
29	SI_CA_Week_0	0	5.34	0.15**	-0.11	5.69
29	SI_FMDV_Week_8	7	4.90	-0.10*	0.12	5.03
29	SI_FMDV_Week_10	0	4.38	-0.11**	0.08	4.61

1. Chr: the chromosome number of the QTL. Underlined if 2 QTL model.

2. Trait: each trait is shown as follows: trait type (IgG1; IgG2; SI_FMDV = T cell proliferation to the FMDV peptide; SI_CA = T cell proliferation to Concanavalin A), followed by week post immunisation.

3. cM: the position the QTL is on the chromosome, in centiMorgans.

4. F: the F-statistic for each QTL. Significance level: all are at least 5% chromosome wide, * = p < 1% chromosome wide, **= p < 5% genome wide and ***= p < 1% genome wide.

5. "a" and "d" are the additive and dominance effect, respectively, of each QTL, * = p < 5%, **= p < 1% and ***= p < 0.01%.

6. Var%: the phenotypic variance explained by the QTL.

Clusters of related traits have been boxed, 6 in total

Each of the four trait types (IgG1 response; IgG 2 response; T cell response to FMDV and T cell response to Con A), were clearly under polygenic control (Table 1 and 2), and no QTL were found that controlled all traits. Of the 77 QTL detected, the majority (51) played a role in determining the FMDV15 specific antibody responses, whereas fewer (26) controlled the T cell response to FMDV15 and Con A.

**Table 2:** Chromosomal positions of all QTL

		BTA number^3^												
**Trait^1^**	**Week^2 ^**	**9**	**11**	**23**	**25**	**13**	**26**	**4**	**21**	**20**	**24**	**2**		

IgG1	0	x^4^	x	x	x									
	1			x	x	x	x	x						
	2			x	x				x	x				
	4									x				
	8							x		x	x			
	10			x				x		x	x	x		

		14	18	25	3	16	19	27	20	23	7	12	15	24

IgG2	0	x												
	1		x	x										
	2			x	x	x	x							
	4			x			x	x	x	x				
	8						x		x	x	x	x	x	
	10								x	x				x

		4	20	6	7	29	9							

T-cell FMDV	0	x	x	x										
	4				x									
	8			xx		x								
	10			x		x	x							

		29	6	5	24	19	25	7	18					

T-cell ConA	0	x	x	xx										
	4				x									
	8					x								
	10			x		x	x	x	x					

1. Trait = immune response type to the FMDV15 peptide

2. Week post immunisation

3. Bos Taurus Autosome (BTA) number

4. Each 'X' represents the presence of a QTL, 'XX' represents 2 QTL. All are at least 5% chromosome wide significant

(AUC results are omitted as they are representative of the overall response across time.)

**Figure 3:**
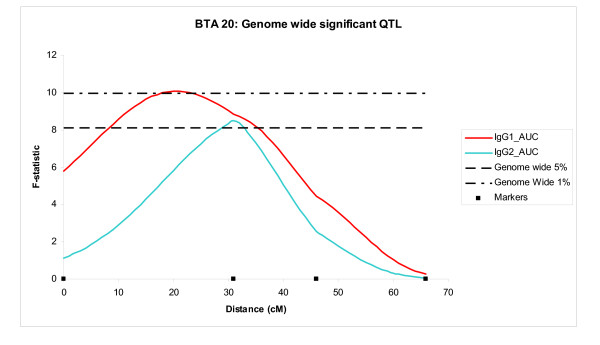
**BTA23: Significant QTL**. *F*-statistic profile and support intervals for IgG1 and IgG2 responses (AUC = area under curve) elicited by FMDV15 peptide, located on chromosome 23. The constant horizontal line represents the threshold of the 1% chromosome wide significance (*F *= 5.90). Traits are described as Table 1.

**Figure 4:**
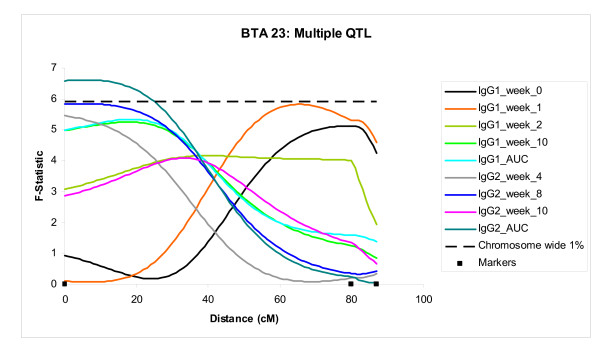
**BTA20: Significant QTL**. *F*-statistic profile for IgG1 and IgG2 responses (AUC = area under curve) elicited by FMDV15 peptide, located on chromosome 20. The dashed horizontal line represents the threshold of the 1% genome wide significance (*F *= 9.94). The dot-dashed line represents the threshold of the 5% genome wide significance (*F = 8*.10). Traits are described as Table 1.

With few exceptions, different QTL were detected at different time points. The majority of QTL appeared to be specific for a given trait type or for early or late time points, suggesting that T cell and antibody response must be, in the main, under the control of different genes (Table 2). A few chromosomal regions appeared to control more than one type of response in particular QTL on BTA 20 (Figure 3 and Table 2), BTA 23 (Figure 4) and BTA 25 (all 3 BTA shown in Table 1) impacted on the greatest number of traits (9, 9 and 7 respectively). QTL on BTA 4, 19, 20 and 23 all played a role in both the primary and secondary responses, whereas QTL on BTA 20, 23, 24 and 25 all affected the FMDV15 specific IgG1 and IgG2 at several time points, although the QTL located on BTA 25 specifically influenced the primary IgG1 and IgG2 responses and not the secondary antibody responses. In contrast the QTL on BTA 24 was detected for the secondary responses of both antibody isotypes but not the primary responses. Interestingly, the QTL on BTA 23 influenced earlier time points for the IgG1 response, and not the IgG2 response (Table 1 and Figure 4). The QTL with highest additive effect (p < 0.01) was identified on BTA 23 associated with the IgG2 response at week 10, where the additive effect of the Holstein allele was an increase of 10.5 μg/ml. IgG2 responses in week 8 on BTA 12 and 15, both showed large dominance effects of 17.4 μg/ml and 11.0 μg/ml (p < 0.01 and p < 0.1, respectively).

Very little overlap was observed in the chromosomal regions controlling the T cell response to the FMDV 15 immunising antigen, and the T cell response to the T cell mitogen, ConA, with the exception of QTL on BTA 6 and BTA 29. In addition, very little overlap was seen between QTL controlling the T cell responses and the antibody responses, with the exception of the QTL on BTA 20, which appeared to play a role at several time points for both antibody isotypes as well as the T cell response at the initial immunisation time point. Indeed the QTL with the highest F value (10.07; 1% genome wide significance) in the study was for the AUC for the anti-FMDV15 IgG1 response, and was located on BTA 20, where the clusters of QTL controlling IgG1 and IgG2 traits are also located. This QTL alone accounted for 9.8% of the variance for the overall IgG1 response. Other highly significant QTL included a QTL on BTA 24 for the IgG1 response at week 8 and T cell response to Con A at initial immunisation on BTA 6 (both 5% genome wide significance).

### 2 QTL Model

**Figure 5:**
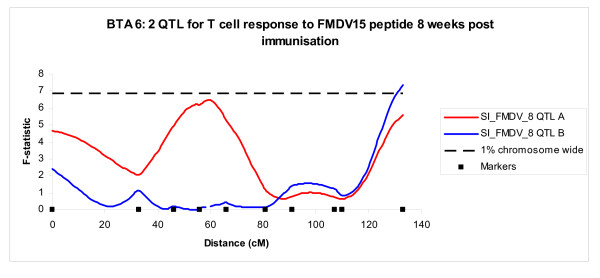
**BTA6: 2 Significant QTL**. F-statistic profiles for two QTL on BTA6 for the T cell proliferation to FMDV15 peptide at week 8. The peak of QTL A occurs at 59 cM. Following the addition of QTL A as a background effect, QTL B was revealed. The peak of QTL B is at 133 cM. The horizontal line is the 1% chromosome wide significance level (F = 6.85). Traits are described as Table 1.

Following the initial analysis, each QTL was added as background effect and 2 QTL models were run. This analysis identified 3 significant 2 QTL models (Table 1). These included the T cell response to Con A at Week 0 on BTA 5; T cell responses to the FMDV15 peptide at Week 8 (Figure 5) and AUC, both on BTA 6. Each of these pairs of QTL on single chromosomes had at least two markers separating them.

### Clusters

Although a few chromosomes (BTA 2, 3, 11, 12, 13, 14, 26 and 27) only appeared to have QTL affecting an individual immune-related trait at one time point, several chromosomes had QTL affecting a trait across time. All chromosomes with greater than 3 QTL for the same trait across time (i.e. IgG1 weeks: 0, 2 and 4) with overlapping CI are shown with outline boxes in Table 1 and are referred to as "clusters". There were 6 such clusters located on BTA 4, 5, 6, 20, 23 and 25 (Table 1; Figure 3, 4 and Table 2). The most significant QTL in the study was located within the clusters of QTL for IgG1 and IgG2 responses on BTA 20 (Table 1). The greatest phenotypic variation explained within this cluster for the IgG1 response was 9.82% and 8.41% for the IgG2 response. This region also influenced the T cell response to FMDV15 at the time of immunisation. BTA 23 contained two QTL clusters for IgG1 and IgG2 responses. All of the clusters, with the exceptions of the clusters on BTA 4 and BTA 5, had additive effects showing Holstein alleles increased the phenotype in a favourable direction.

## Discussion

This study has revealed that both the humoral and cell mediated immune response to a relatively simple 40-mer peptide are under the control of a considerable number of chromosomal loci. The phenotypes described in the present study were all obtained using a standardised experimental protocol and a uniform dose of a specific antigen. This study is also unique in that it was possible to measure both a primary and secondary immune response across time and thus detect QTL that influenced different phases of the immune response. These considerations may, at least in part, explain the large number of QTL detected. The outcomes of disease resistance or vaccine induced protection are likely to depend on a range of immune-related mechanisms. The present study concentrated on some of these underlying traits, namely antibody and cell mediated immune responses. Although these have been shown to play a role in host protection against FMDV [5-8], considerable animal-to-animal variation is observed and no immunological parameter correlates entirely with protection. Our study suggests that host genetics may be an important factor underpinning these discrepancies and further research on the relationships between host genotypes, accurately determined phenotypes in experimental studies, such as ours, and protection and disease outcome as measured in field studies are clearly warranted.

Unexpectedly, a number of QTL were detected for the 0 time point for both antibody and T cell proliferation. It is difficult to interpret these results as the animals were naïve to the FMDV peptide. In a previous study it was noted that the control values for T cell proliferation were under genetic control [14], and thus the QTL for the T cell proliferation to the FMDV peptide at time 0, may be related to the inherent ability of T cells in culture to proliferate. Measurements of cellular immunity are inherently more difficult to conduct than measurements of humoral immunity, partly because cell based assays have to be conducted with fresh cells. Generally T cell proliferation is measured by purifying peripheral blood mononuclear cells (PBMC) and measuring the response at day 5 of culture [17]. For the work reported here a high-throughput system had to be devised to measure the cellular immune response to the FMDV peptide in large numbers of animals, which involved whole blood cultured for 6 days which may have resulted in greater variation compared to the proliferation response by purified peripheral blood mononuclear cells. A further factor that may explain the high 0 time values is that the FMDV peptide contains an "RGD" motif which enables the FMDV virus to bind to integrins on the cell surface to facilitate virus entry [18]. Fluorescently labelled FMDV peptide has been shown to bind to a wide variety of cells (Glass, unpublished observation) potentially through integrin binding. In addition it is possible that there are soluble molecules in serum that bind to the "RGD" motif in the peptide coated on the ELISA plate: this may have caused a "background" non-FMDV specific binding. Thus possibly the QTL might be reflect variants in integrin molecules. The genes for the main integrin receptor by FMDV for entry into cells are ITGAV and ITGB6 on BTA 2 (unpublished observations) which does not appear to influence the values for the 0 and 1 time points. Nonetheless, the values seen at time 0, for both antibody and proliferation, are orders of magnitude lower than the adaptive immune responses generated to the peptide, and for the main part the time 0 values did not correlate with the responses observed at later time points.

One of the key genome regions controlling immune responses is the MHC locus [19]. Our study provides further support for the relative role of the MHC and adds further confidence to the QTL discovered. However the current study has shown that even the response to a relatively simple antigen is complex and controlled by many loci.

Throughout the study the cohort of the animals was significant (the cohorts correspond to the year of birth of the animals). Environmental sources of variation were minimised as far as possible, including husbandry, food, food supplements and farm. Cohort was, however, found to be significant in the same herd for response to a BRSV vaccine [13], a mastitis causing pathogen [14] as well as in meat and milk traits [15]. Weight and age at vaccination were also significant throughout the study. Lighter animals had higher T cell responses in comparison to heavier animals, suggesting that breeding for heavier animals may have a detrimental effect on an animal's ability to fight infection. Linear regression of IgG1 AUC against age showed that the older animals had significantly higher immune responses (data not shown). This suggests that even in older animals the immune system may still be developing, although possibly these changes may be hormonally related as all the animals in this study were female and there are clear gender and hormone related differences in immunity [20]. The increase in response is not a result of exposure to FMDV, unlike the situation for endemic pathogens such as BRSV which could conceivably influence the response to BRSV derived antibody. Age was also a significant factor in the IgG1 and IgG2 response to a BRSV vaccine in this experimental herd [13]. However, the animals were much younger than in the present study.

Significant line effects have previously been shown for response to a BRSV vaccination [13], but not response to *Staph. aureus *[14]. The current study also did not find any line effects. It is possible that the founder breeds, Holstein and Charolais, have similar immune responses to the FMDV15 peptide, although this has not been formally tested. *Villa-Angulo et al *[21] have shown that Holstein and Charolais cattle breeds have highly correlated haplotype blocks, thus it is feasible that the similar immune response seen in this study is due to the two breeds being highly related. However, within the QTL study 6 of the 8 QTL clusters had increased phenotypes resulting from Holstein alleles, indicating that there might be breed differences in the pathways used to initiate and maintain a response to the FMDV15 peptide. Sire effects have previously been demonstrated in this herd, both in the immune response to a BRSV vaccine [13] and also to *Staph. aureus *[14]. In the present study sire effects were only significant for the IgG2 response to the FMDV15 peptide. Dam was only sporadically significant without any obvious patterns to explain the weeks it appeared significant. However, the number of calves per dam was small, so it is unlikely that enough statistical power existed to detect dam effects.

The immune response is a complex trait and across time different factors will play a role in affecting the level of a particular response, for example, the initiation of an immune response involves components of innate immunity such as macrophages and dendritic cells which signal alarm to the adaptive immune system which takes longer to respond to immunisation and invasion by pathogens. In addition, the primary response is under the influence of different factors from the secondary response, which is generally of greater magnitude and longer duration. Nonetheless, several QTL which influenced the same traits were detected across time, suggesting that there may be gene(s) that impact on both the primary and secondary responses. Thus genetic factors may underlie some of the significant correlations across time and within traits (Additional File 1). The highest correlations observed were within traits and several chromosomes contain QTL for the same trait at different time points. This suggests that responses at early time points can be predictive of those at later time points, information that may be useful in QTL studies where it is not possible to make serial measurements. However, the data presented here also suggests that there are several loci which have more specific effects either at single time points or on specific immune traits, which may reflect the different cell types and factors operating at different phases of the immune response.

Inter-trait correlations were observed between the IgG1 and IgG2 responses (Additional File 1), although for the most part the QTL controlling these traits did not coincide on the same chromosomes, with the notable exceptions of the QTL on BTA 23, the 95% confidence interval of which harboured the *BoLA *locus, and BTA 20. Both of these chromosomes may have genes that control both types of antibody response across time. All animals had considerably higher IgG1 than IgG2 responses, with a proportion having no detectable IgG2 response at any time point [11]. The QTL with the greatest influence on the overall IgG1 responses were BTA 4, 20, 23 and 24 which together accounted for over 25% of the variance. Apart from BTA 20 and 23, two different regions on BTA 15 and 25 were also linked to the overall IgG2 response, and these 4 regions also accounted for over 25% of the variance for overall IgG2 response. In cattle it appears that IgG1 is associated with type 2 T cell responses and IgG2 with type 1 [22]. Thus regions controlling the differing responses may harbour genes that influence the balance between Th1 and Th2 responses; plausible candidate genes under QTL include IL18 on BTA 15 and IL4R on BTA 25.

The T cell responses to the ConA mitogen and the FMDV15 peptide were also correlated, in particular the responses at the same time points. This was unexpected as immunisation with the FMDV15 peptide should not influence the responses to ConA, as the latter is a T cell mitogen. One possible explanation may be that the T cells stimulated by the FMDV peptide become more responsive to T cell mitogens, indeed proliferation was observed in the presence of medium alone and increased across time, suggesting that immunisation may have activated the overall T cell population. However, with the exception of BTA 6, 7 and 29, no co-incident QTL for T cell response to ConA and FMDV15 were observed. Further, as no correlations were detected in the responses of the T cell proliferation compared to IgG levels, there seems not to have been a cellular or humoral bias in this study.

Interestingly, every QTL cluster, with the exception of the cluster on BTA 25, contained at least one QTL above the 1% chromosome-wide significance level. Thus the clusters appear to highlight regions of the bovine genome that are highly associated with the immune response to the FMDV15 peptide. The two clusters containing the QTL associated with the greatest number of traits were located on BTA 20 (Table 1; Figure 3) and 23 (Table 1; Figure 4; both included in Table 2). The cluster located on the telomeric region of chromosome 20 has highly significant dominant effects, suggesting that over-dominance may have a role to play in these QTL. Further, this region has also been associated with a number of diseases, including resistance to *Mycobacterium avium *ssp Paratuberculosis the causative bacterium of Johne's disease [23], bovine keratoconjunctivitis (pinkeye) [24] and respiratory disease and pododermatitis (footrot) [25]. Taken together with the data presented here this suggests that gene(s) located in this region may control the response to several bacterial and viral pathogens, possibly by influencing antibody production. The current data suggest that the gene(s) underlying this QTL impact on the antibody responses across time. Further research to identify the gene(s) underlying these associations is warranted as they may represent genes for "generalised immune competence" or resistance to a broad range of pathogens.

The QTL cluster located on BTA 23 includes the bovine *MHC *(*BoLA*) region [26]. However, few markers were spaced across BTA 23, and the QTL CIs covered the whole of BTA 23. Our previously study using the same population of animals showed that the highly polymorphic *BoLA DRB3 *gene is significantly associated with the response to FMDV15 [11] in this herd and earlier studies have indicated that *DRB3 *polymorphisms are also linked to protection against viral challenge following immunisation with FMDV15 and similar peptides [9]. It is therefore likely that the causal genes underlying the part of the BTA 23 QTL effect may be coded for within the BoLA region. However, there are at least 154 predicted genes in this region [27], which may make it difficult to identify the causative mutations. Other QTL studies conducted on this herd, for traits such as meat quality [15], coat colour [16] and temperament [28] also have QTL which overlap (+/- 10cM) with QTL discovered in the current study. QTL for meat quality overlapped with 6 QTL in the current study (BTA: 6, 13, 15, 16, 19 and 25), QTL for temperament also had 6 overlapping QTL (BTA: 4, 7, 9, 18, 20 and 25) with the same QTL on BTA 25 appearing in all 3 studies, suggesting a major gene/pathway may exist below these QTL which affects, not only the immune response, but the quality of meat and the temperament of the animals. Only one QTL overlapped with QTL from the coat colour study, located on BTA 5. No clear pattern appears in the overlapping QTL, with exception for the QTL on BTA 25.

The QTL cluster on BTA 6 overlaps with a QTL that is associated with a mastitis related trait [29]. Recently *Jann et al *[30] have identified the toll like receptor (TLR) cluster of TLR1, 6 and 10 genes as the most likely candidate genes within this region on BTA 6. TLRs play an important role in both innate and adaptive immunity [31]. *Villa-Angulo et al *[21] demonstrated that BTA 6 has haplotype blocks that are similar (r^2 ^= 0.61) between the breeds used in the current study, thus the genes in both breeds are likely to be in the same regions and orders in the cluster of TLR 1, 6 and 10.

## Conclusion

Although a variety of traits in diseased and clinically healthy animals have been used to identify QTL in livestock [31]. This study focused specifically on immune responses across time following a specific immunisation procedure to identify QTL controlling defined immune responses. The density of markers and numbers of animals used is not sufficient to fine map the QTL regions identified, however, further work is warranted especially to refine the QTL clusters, which appear to be the most promising regions. With the availability of the bovine genome sequence [27], the bovine HapMap [32] and dense bovine SNP arrays [33] unprecedented opportunities are now available to identify genes and pathways underlying immune-related traits. The QTL discovered in this study may ultimately reveal novel targets for both the selection of disease resistant livestock as well as for the improvement of vaccines in their efficacy.

## Methods

### Animals

A total of 501 second generation cross bred animals were produced in the Roslin Bovine Genome (RoBoGen) herd. Pure-bred Charolais sires (8) were mated to Holstein dams (221) to produce the F1 (8 male, 134 female) and 8 F1 sires were mated to F1 heifers to create the F2 group within the second generation. The founder Charolais sires were mated to F1 heifers to produce a Charolais backcross (CB1) and F1 sires were mated to pure bred Holstein heifers to create a Holstein backcross (HB1). Immune response measurements were collected from the 195 second generation cross heifers (121 from the F2, 43 from the HB1 and 31 from CB1). All female calves were weaned by 36 h, segregated from the rest of the herd and raised indoors, initially on milk-replacer then weaned early onto a propriety compound diet. The age of the first immunisation with the FMDV15 peptide ranged from 469-609 days.

### Immunisation and sampling

The FMDV15 peptide was chemically synthesized using an ABI 431A peptide synthesiser with FMOC chemistry. Following deprotection and cleavage, it was purified by preparative reverse phase HPLC (Beckman Coulter System Gold HPLC using a Phenomenex Luna C18 column). The peptide consisted of two separate regions (residues 141 to 158 and 200 to 213) of the virus coat protein (VP1) from the O1 Kaufbeuren strain of foot-and-mouth disease virus [4]. The female F2 and backcross heifers were immunised subcutaneously with 1 mg FMDV15 peptide/animal emulsified in Freund's incomplete adjuvant at week 0, followed by a boost of 100 μg FMDV15 peptide/animal at week 6. Whole blood samples were collected by jugular venipuncture from all of the female F2 and backcross heifers at weeks 0, 1, 2, 4, 8 and 10, post immunisation for IgG analysis (see below) and at weeks 0, 4, 8 and 10 post immunisation for T-cell measurements (see below). For the IgG analysis, the blood samples were allowed to clot, and serum collected and stored at -20°C until they were tested for the T cell analysis, blood was collected aseptically into heparin tubes. All experimental protocols were authorised under the UK Animals (Scientific Procedures) Act, 1986.

### Phenotypic Data

#### Whole blood T-cell Proliferation assay

The T-cell proliferation assay was carried out essentially as described by Glass et al 1990 [17] with the exception that whole blood was used. Whole blood (150 μl) was diluted with 750 μl RPMI 1640 supplemented with 25 mM HEPES, 2 mM glutamine, 10% foetal calf serum, 5 × 10^-5 ^M 2-mercaptoethanol either alone as the negative control, 10 μg/ml concanavalin A (ConA) (Sigma, UK) as a positive control or 2.0 μg/ml FMDV15 peptide (all final concentrations).

Quadruplicate cultures were incubated at 37°C with 5% CO_2 _for 6 days. For the last 6 hours the cells were labelled with 0.037MBq^3^H-Thymidine per well (GE Healthcare, UK) and uptake assessed by liquid scintillation counting using a 1450 Microbeta (Wallac, now PerkinElmer). The results were expressed as counts per minute of ^3^H-Thymidine incorporation (mean of quadruplicates).

#### ELISA for detection of FMDV15-specific IgG1and IgG2

FMDV15 peptide specific ELISAs were performed to measure IgG1 and IgG2 isotypes as detailed in Baxter *et al *2009 [11]. ELISA tests were conducted on the samples from all three cohorts, over a short period following the final sampling, to minimise technical variation. Briefly, Immunolon 2HB plates (Dynex Technologies) were coated with 100 μl of 1 μg/ml FMDV15 peptide in carbonate/bicarbonate buffer (Sigma), serum samples were added at a dilution predetermined to fit within the standard curve (see below) concentrations. 100 μl HPR-conjugated sheep anti bovine IgG1 (Cat. No. A10-116P, pre-optimised at 1/20,000 dilution) or IgG2 (Cat. No.A10-116P, pre-optimsed at 1/25,000 dilution) (Bethyl Montgomery, Texas, USA) were added as the secondary antibodies and colour developed with Sure Blue Reserve tetramethylbenzidine (TMB) microwell peroxidase substrate 1 component (KPL). Optical density was measured at 450 nm on a Victor^2 ^1420 Multilabel counter (Wallac). Seven serial dilutions of bovine reference serum (Bethyl), containing known concentrations of IgG1 and IgG2 in carbonate/bicarbonate buffer were included on each plate which enabled the IgG1 and IgG2 concentrations to be calculated from simple linear regression (Genstat [34]).

### Statistical Analysis

Stimulation Indexes (SI) were calculated for the T-cell proliferation to the FMDV15 peptide (A_Y_) and ConA (A_Z_), where SI = A_Y or Z_/B_x _and B_x _was the negative control.

The IgG1 and IgG2 concentrations and the T cell SI for ConA and FMDV15 were normalised to obtain a normal distribution and constant variance (log_10_). Area under the curve (AUC) for each of these four traits was also calculated (using the trapezoidal rule [35]) to provide a single trait that reflected the overall response of the normalised data.

REML (REsidual Maximum Likelihood) was used, within Genstat [34], to determine which factors were significant within the herd. Sex was omitted from the model as all of the phenotyped animals in this study were female. The final model included sire and dam as random effects; the other effects within the model were, with appropriate degrees of freedom (df), *line *(F2, CB1, HB1; 2 d.f) and *cohort *- corresponding to the 3 years of the study (1, 2, 3; 2 d.f). *Age *(age at vaccination; 89 d.f) and *calculated weight *(weights of animals at initial vaccination date calculated from regression of animal weights at other time points pre and post vaccination; 193 d.f) were both covariates.

Thus the final REML model was:

$${\text{Y}}_{\text{jqmanfp}}=\mu +\beta_{\text{j}}+{\text{C}}_{\text{q}}+\gamma_{\text{m}}+{\text{W}}_{\text{a}}+{\text{u}}_{\text{jqman}}+{\text{g}}_{\text{jqmanf}}+{\text{e}}_{\text{jqmanfp}}$$

Where: Y_jqmanfp _is the observed value of the phenotypic trait; μ, population mean; β_j_, the fixed effect of line j (j = 1, 2, 3); C_q_, the fixed effect of cohort q (q = 1,2,3); γ_m_, the linear covariate of age at vaccination m (m = d469-d609); W_a_, the linear covariate of weight a (a = 361-744 Kg); u_jqman_, the random effect of sire; g_jqmanf_, the random effect of dam; e_jqmanfp_, the residual error p, p ~ N(0,Iσ^2^_p_). The residual variance from the model was used to calculate correlations.

A chi squared test (with 1 df), using the difference of the deviance (-2* log likelihood) of the sire and dam, either in or out of the REML model, was used to calculate the significance of the sire and dam. Wald tests were used to determine the significance of the other effects.

### Genetic Markers

Standard phenol-chloroform methods were used to extract DNA from blood samples [36]. A panel of microsatellite markers were genotyped across all individuals in the herd, with a total of 165 microsatellite markers distributed across all 29 autosomes [15]. All of the genotypes were stored in the database ResSpecies [37] and used to build linkage maps with CRIMAP 2.4 [38]. The maps were compared to the latest bovine linkage map [39]. Once compared for consistency, the maps constructed using CRIMAP, were used to conduct the QTL analysis for the immune-related traits (Additional File 4).

### QTL Analysis

The 24 traits tested in the QTL analysis were: FMDV15 specific IgG1 and IgG2 concentrations at weeks 0, 1, 2, 4, 8, 10 and AUC; and T-cell proliferation to ConA and the FMDV15 peptide at weeks 0, 4, 8, 10 and AUC.

GridQTL [40], internet based software, was used for the QTL analysis. The F2 and backcross module was used which assumes that founder lines are fixed for alternative alleles at QTL loci (although they can be segregating at markers) and implements a least squares analysis. All effects found significant from the previous analysis using REML were included in the model, thus for each time point only significant effects were added to the QTL model. Information content (IC) along the linkage maps was also calculated by the program. Both single and two QTL models with additive and dominance effects were fitted at 1 cM intervals along the autosomes (N = 29), using the sex averaged genetic map (Additional File 4). By setting the 2 founder breeds as: breed 1 = Holstein and breed 2 = Charolais, the positive or negative sign of the additive effects indicated that the Holstein or Charolais allele, respectively, increased the trait values. When the dominance effect had the same sign as the additive effect, this indicated dominance of the Holstein allele over the Charolais allele, whereas if the signs of the additive and dominance effects were opposite, the Charolais allele was dominant.

In total QTL were sought for 24 traits using the 165 microsatellite markers spread across all 29 autosomes of the bovine genome. Significance thresholds were calculated by permutation analysis with 1000 permutations [41]. Four significance levels were used: chromosome-wide 5% and 1% and genome-wide 5% and 1%.

### Refining QTL

The QTL detected at the 5% chromosome-wide significance level and above were included in the model and the genome rescanned for further QTL. By adding the initial QTL as background effects, the variance caused by them is removed, thus potentially revealing previously undetected QTL. In cases where more than one QTL was found for the same trait on the same chromosome, a 2 QTL model was performed by fitting two QTL simultaneously and re-analysing the data. A forward and backward selection interval mapping approach was used to check whether QTL moved significantly, relative to other QTL of the same trait [42], however none did. These refining methods were repeated, until no further QTL were detected. Finally, bootstrap analysis was performed, using 1000 repeat samples, for all chromosomes where a significant QTL was detected at the 5% chromosome-wide threshold [43] to estimate the 95% confidence intervals for location of the QTL, except for the 2 QTL models which were not bootstrapped. The confidence intervals for 2 QTL models were calculated by placing one of the QTL in the model to calculate the other QTL's confidence interval. This would be repeated with the other QTL and the lower and upper confidence intervals would be used to set the confidence interval.

## Authors' contributions

RJL prepared and drafted the manuscript, conducted the statistical analysis, designed and ran the QTL analysis. SCC conducted all of the lab based work. SAK: helped to edit the manuscript. EJG conceived the initial idea of the study, and participated in its design and coordination and helped to draft the manuscript. JLW initiated and established the overall project design and was responsible for creating the cross-bred animal population used, had an input to design of the immune studies and helped in editing the manuscript. All authors read and approved the final manuscript.

## Supplementary Material

Additional file 1 **Correlation matrix of traits**. Residuals for the REML model were used to calculate the correlations between each week and trait. r^2 ^values that are +/- 0.2 are coloured grey and are significant to p < 0.05 Click here for file

Additional file 2 **Factors used in the REML model and their significance**. Only p-values <0.05 are shown for each week for all four traits. Line was not significant for any trait at any time point. Click here for file

Additional file 3 **Detailed description of QTL located in this study**. Supplementary table showing extra detail of each QTL: 1. Chromosome: the chromosome number of the QTL. Underlined if 2 QTL model. 2. Trait: each trait is shown as follows: trait type (IgG1; IgG2; SI_FMDV = T cell proliferation to the FMDV peptide; SI_CA = T cell proliferation to Concanavalin A), followed by week post immunisation. 3. cM: the position the QTL is on the chromosome, in centiMorgans. 4. F: the F-statistic for each QTL. Significance level: all are at least 5% chromosome wide, * = p < 1% chromosome wide, **= p < 5% genome wide and ***= p < 1% genome wide. 5. Flanking markers of each QTL peak. 6. The 95% confidence intervals of each QTL. 7. "a" and "d" are the additive and dominance effect, respectively, of each QTL, * = p < 5%, **= p < 1% and ***= p < 0.01%. 8. "a/SD" and "d/SD" are the standard deviation units for the additive and dominance effects, respectively. Click here for file

Additional file 4 **Linkage map**. Marker distances (cM Kosambi) are shown for the sex-average maps built for the Charolais × Holstein population used in this study. Click here for file
