# Supplementary figures and images for: Quantitative trait loci for variation in immune response to a Foot-and-Mouth Disease virus peptide
Source: BMC Genet. 2010 Dec 7;11:107. doi: 10.1186/1471-2156-11-107 (PMC3019142; doi:10.1186/1471-2156-11-107)

## Slide 1
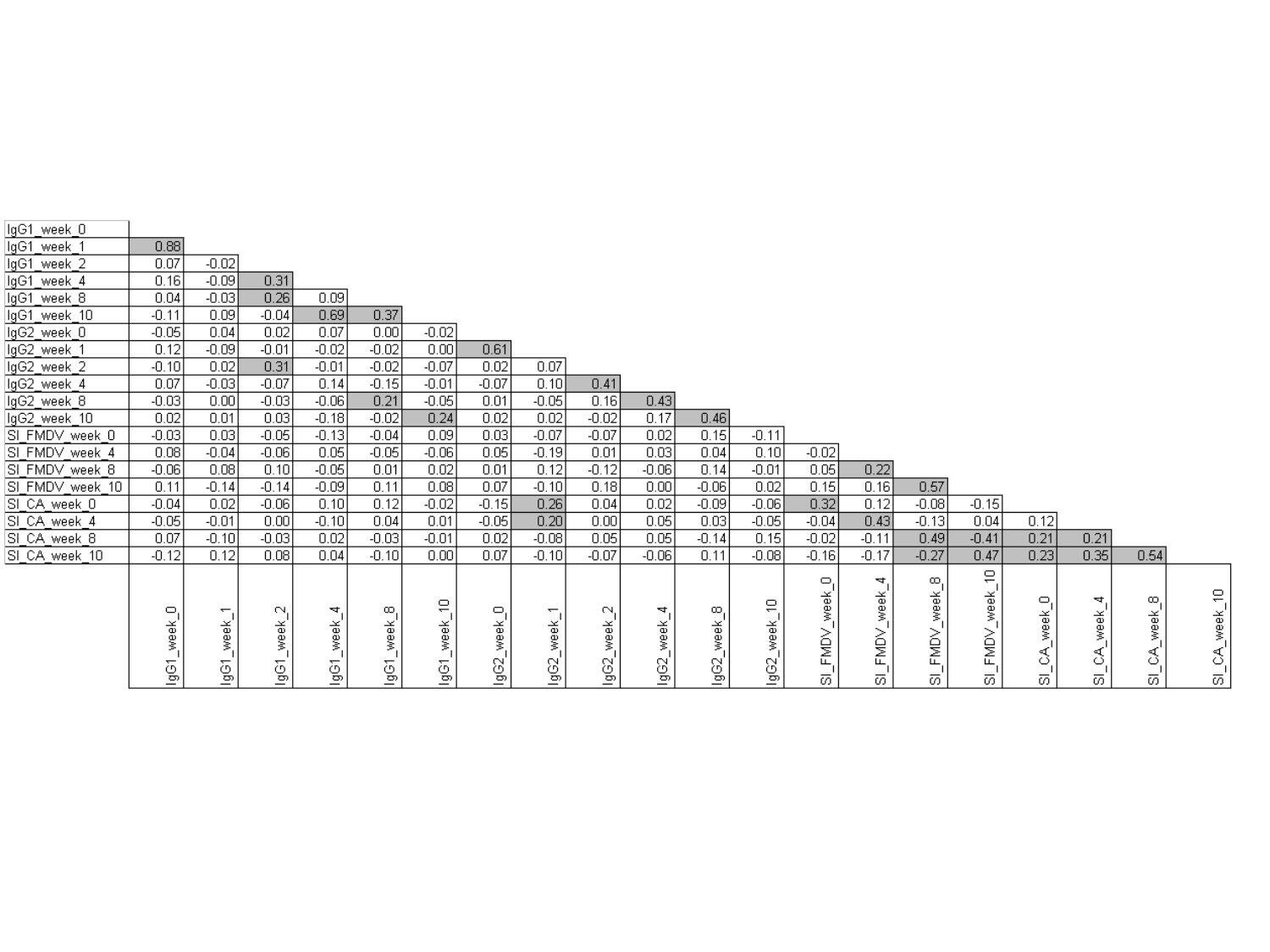

Supplement: Additional file 1 — Correlation matrix of traits. Residuals for the REML model were used to calculate the correlations between each week and trait. r2 values that are +/- 0.2 are coloured grey and are significant to p < 0.05 [file 1471-2156-11-107-S1.PPT]

## Slide 1
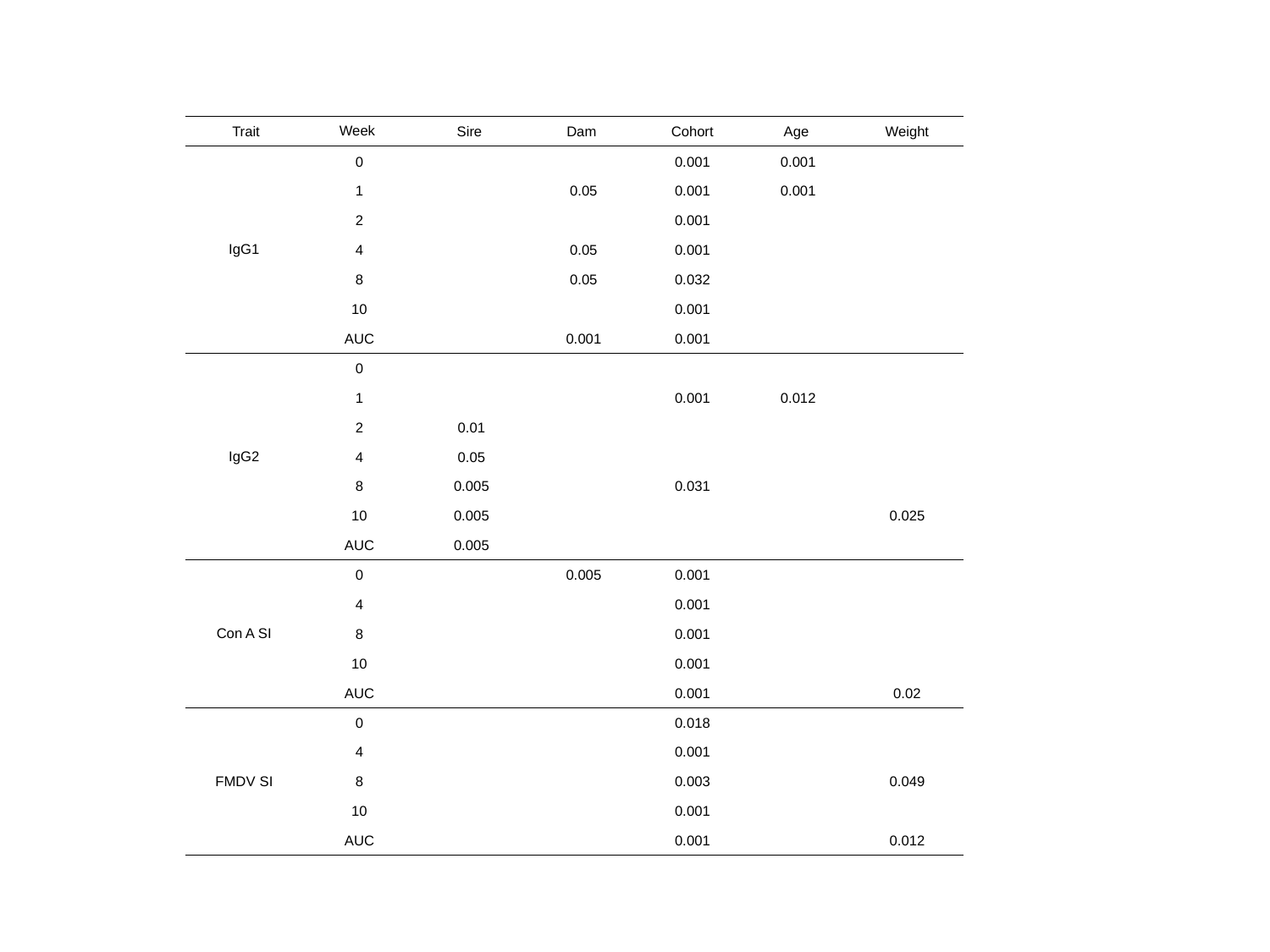

| Trait | Week | Sire | Dam | Cohort | Age | Weight |
| --- | --- | --- | --- | --- | --- | --- |
| IgG1 | 0 | | | 0.001 | 0.001 | |
| | 1 | | 0.05 | 0.001 | 0.001 | |
| | 2 | | | 0.001 | | |
| | 4 | | 0.05 | 0.001 | | |
| | 8 | | 0.05 | 0.032 | | |
| | 10 | | | 0.001 | | |
| | AUC | | 0.001 | 0.001 | | |
| IgG2 | 0 | | | | | |
| | 1 | | | 0.001 | 0.012 | |
| | 2 | 0.01 | | | | |
| | 4 | 0.05 | | | | |
| | 8 | 0.005 | | 0.031 | | |
| | 10 | 0.005 | | | | 0.025 |
| | AUC | 0.005 | | | | |
| Con A SI | 0 | | 0.005 | 0.001 | | |
| | 4 | | | 0.001 | | |
| | 8 | | | 0.001 | | |
| | 10 | | | 0.001 | | |
| | AUC | | | 0.001 | | 0.02 |
| FMDV SI | 0 | | | 0.018 | | |
| | 4 | | | 0.001 | | |
| | 8 | | | 0.003 | | 0.049 |
| | 10 | | | 0.001 | | |
| | AUC | | | 0.001 | | 0.012 |

Supplement: Additional file 2 — Factors used in the REML model and their significance. Only p-values <0.05 are shown for each week for all four traits. Line was not significant for any trait at any time point. [file 1471-2156-11-107-S2.PPT]

## Slide 1
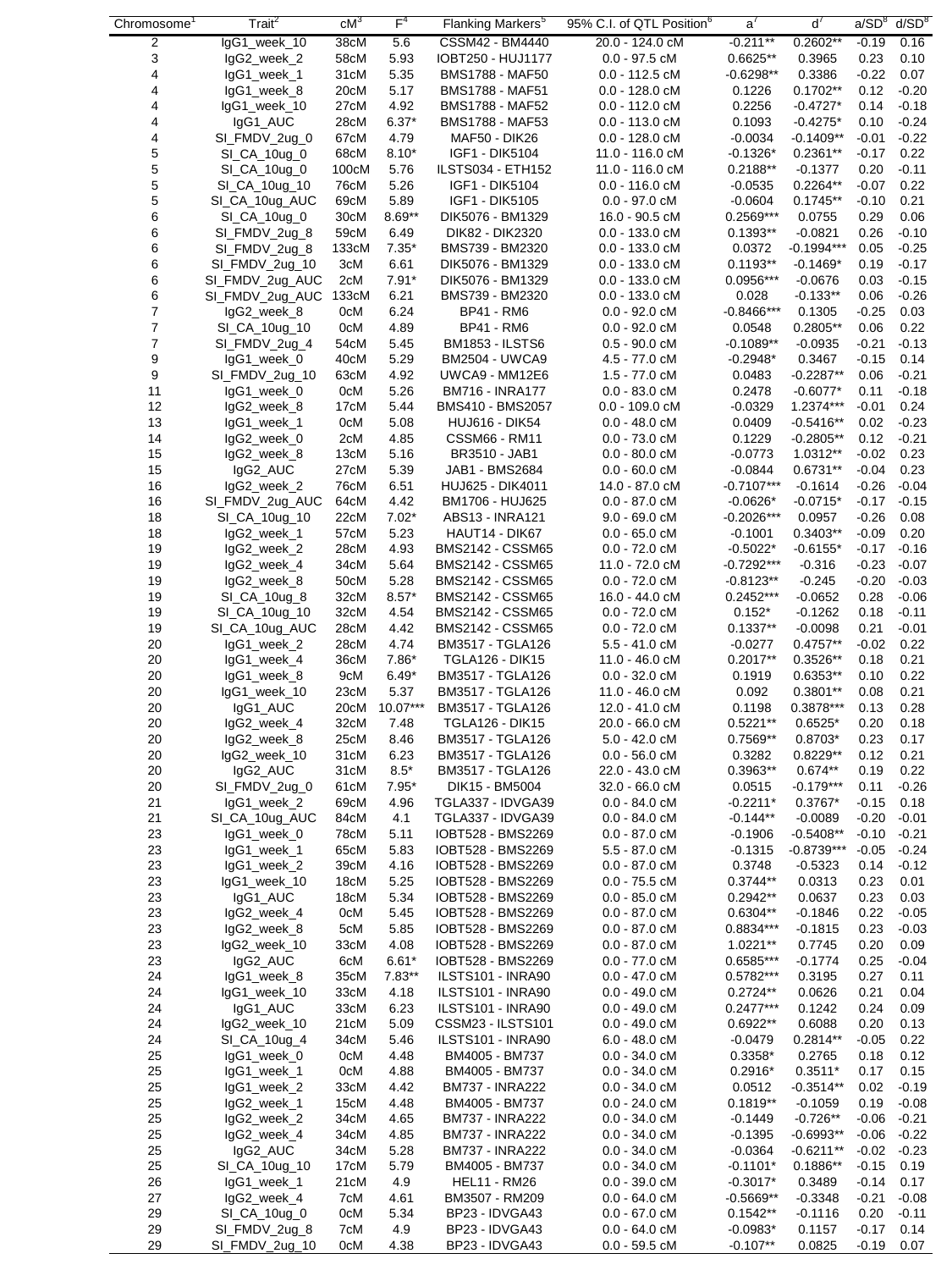

Supplement: Additional file 3 — Detailed description of QTL located in this study. Supplementary table showing extra detail of each QTL: 1. Chromosome: the chromosome number of the QTL. Underlined if 2 QTL model. 2. Trait: each trait is shown as follows: trait type (IgG1; IgG2; SI_FMDV = T cell proliferation to the FMDV peptide; SI_CA = T cell proliferation to Concanavalin A), followed by week post immunisation. 3. cM: the position the QTL is on the chromosome, in centiMorgans. 4. F: the F-statistic for each QTL. Significance level: all are at least 5% chromosome wide, * = p < 1% chromosome wide, **= p < 5% genome wide and ***= p < 1% genome wide. 5. Flanking markers of each QTL peak. 6. The 95% confidence intervals of each QTL. 7. "a" and "d" are the additive and dominance effect, respectively, of each QTL, * = p < 5%, **= p < 1% and ***= p < 0.01%. 8. "a/SD" and "d/SD" are the standard deviation units for the additive and dominance effects, respectively. [file 1471-2156-11-107-S3.PPT]
