# Supplementary material for: Quantitative trait loci for variation in immune response to a Foot-and-Mouth Disease virus peptide
Source: BMC Genet. 2010 Dec 7;11:107. doi: 10.1186/1471-2156-11-107 (PMC3019142; doi:10.1186/1471-2156-11-107)
Supplement: Additional file 4 — Linkage map. Marker distances (cM Kosambi) are shown for the sex-average maps built for the Charolais × Holstein population used in this study. [file 1471-2156-11-107-S4.DOC]

| BTA | Markers and distances between(cM Kosambi) | BTA | Markers and distances between(cM Kosambi) |
| --- | --- | --- | --- |
| BTA 1 | BM6438 3.3 TGLA49 33.7 BMS4017 14.0 TGLA57 19.6 INRA128 19.4 BM864 29.2 CSSM19 23.1 BMS4044 | BTA 16 | BM121 12.0 TGLA53 15.8 ETH11 28.0 BM719 7.6 BM1706 12.1 HUJ625 12.1 DIK4011 |
| BTA 2 | TGLA431 32.6 CSSM42 22.2 BM4440 26.3 TGLA226 33.4 BM2113 9.8 IDVGA2 | BTA 17 | URB48 28.6 BMS1373 13.2 TGLA231 8.9 IDVGA40 25.8 INRA25 12.5 BM1233 |
| BTA 3 | ILSTS96 24.1 TGLA263 13.2 INRA123 0.1 INRA130 15.0 IOBT250 11.1 HUJ1177 12.4 IDVGA35 22.6 IDVGA27 | BTA 18 | IDVGA31 17.7 ABS13 13.2 INRA121 12.4 HAUT14 25.8 DIK67 |
| BTA 4 | BMS1788 62.3 MAF50 30.0 DIK26 3.8 IDVGA51 16.4 RM88 15.8 MGTG4B | BTA 19 | HEL10 27.6 BMS2142 25.8 CSSM65 18.8 ETH3 |
| BTA 5 | BM6026 18.4 RM103 10.2 BM321 7.8 DIK4782 15.3 BR2936 4.7 ETH10 11.3 IGF1 10.1 DIK5104 19.3 ILSTS034 19.2 ETH152 | BTA 20 | BM3517 31.4 TGLA126 14.7 DIK15 20.6 BM5004 |
| BTA 6 | DIK5076 32.6 BM1329 13.3 DIK1054 10.4 DIK82 9.5 DIK2320 15.3 CSN3 9.4 BP7 16.1 DIK1180 2.9 BMS739 23.9 BM2320 | BTA 21 | HEL5 69.4 TGLA337 15.4 IDVGA39 |
| BTA 7 | BP41 9.5 RM6 44.4 BM1853 30.2 ILSTS6 9.4 INRA53 | BTA 22 | DIK1161 19.5 BMS742 9.0 BM3406 13.3 BM3628 7.0 DIK2443 11.2 HAUT24 10.5 UWCA49 13.1 DIK5307 |
| BTA 8 | IDVGA11 19.7 DIK106 37.3 HUJ174 23.1 HEL9 17.3 DIK74 21.6 CSSM47 | BTA 23 | IOBT528 80.3 BMS2269 7.1 BM1905 |
| BTA 9 | ETH225 16.6 BM2504 23.9 UWCA9 28.3 MM12E6 9.1 INRA84 | BTA 24 | TGLA351 11.1 CSSM23 17.1 ILSTS101 21.5 INRA90 |
| BTA 10 | DIK5169 26.9 BMS528 26.8 TGLA378 12.7 BM888 21.4 CSRM60 8.3 MNB-78 17.5 TGLA272 8.5 CSSM46 10.9 BL1134 | BTA 25 | BM4005 17.5 BM737 17.1 INRA222 |
| BTA 11 | BM716 17.0 INRA177 18.1 ILSTS100 28.5 IDVGA3 11.0 HUJV174 15.0 BMS607 | BTA 26 | ABS12 13.5 HEL11 15.1 RM26 10.4 IOBT730 |
| BTA 12 | BMS410 26.4 BMS2057 17.4 RM162 13.4 BM6404 11.5 DIK4028 13.5 INRA5 12.4 URB054 15.3 INRA209 | BTA 27 | BM3507 15.8 RM209 48.8 BM203 |
| BTA 13 | HUJ616 10.9 DIK54 22.3 ABS10 18.0 DIK93 | BTA 28 | BP23 19.3 IDVGA43 7.6 BMS2658 7.5 IDVGA8 |
| BTA 14 | CSSM66 34.2 RM11 23.8 PZ271 10.3 BM4513 5.4 BM2934 | BTA 29 | TGLA86 21 RM44 10.4 MNB-166 8.3 DIK94 27.8 MNB-101 |
| BTA 15 | BR3510 20.6 JAB1 11.7 BMS2684 5.7 IDVGA10 21.9 DIK2634 20.7 BMS429 |  |  |
